# Can latent heat safely warm blood? – in vitro testing of a portable prototype blood warmer

**DOI:** 10.1186/1471-227X-7-8

**Published:** 2007-08-01

**Authors:** Mark P McEwen, David Roxby

**Affiliations:** 1 Flinders University, Faculty of Science & Engineering, Bedford Park, South Australia 5042; 2 Flinders Medical Centre, Biomedical Engineering Department, Bedford Park, South Australia 5042; 3 Flinders University, School of Medicine, Bedford Park, South Australia 5042; 4 Flinders Medical Centre, Transfusion Service, Bedford Park, South Australia 5042

## Abstract

**Background:**

Trauma/retrieval patients are often in shock and hypothermic. Treatment of such patients usually involves restoring their blood volume with transfusion of blood (stored at 2°C – 6°C) and/or crystalloids or colloids (stored at ambient temperature). Rapid infusion of these cold fluids can worsen or even induce hypothermia in these patients. Warming of intravenous fluids at accident sites has traditionally been difficult due to a lack of suitable portable fluid warmers that are not dependent on mains electrical or battery power. If latent heat, the heat released when a liquid solidifies (an inherently temperature limiting process) can warm intravenous fluids, portable devices without a reliance on electrical energy could be used to reduce the incidence of hypothermia in trauma patients.

**Methods:**

Rapid infusion of red cells into patients was timed to sample typical clinical flow rates.

An approved dry heat blood warmer was compared with a prototype blood warmer using a supercooled liquid latent heat storage material, to warm red cells whilst monitoring inlet and outlet temperatures. To determine the effect of warming on red cell integrity compared to the normal storage lesion of blood, extracellular concentrations of potassium, lactate dehydrogenase and haemoglobin were measured in blood which had been warmed after storage at 2°C – 6°C for 1 to 42 days.

**Results:**

A prototype latent heat fluid warmer consistently warmed red cells from approximately 4°C to approximately 35°C at typical clinical flow rates. Warming of stored blood with latent heat did not affect red cell integrity more than the approved dry heat blood warmer.

**Conclusion:**

Using latent heat as an energy source can satisfactorily warm cold blood or other intravenous fluids to near body temperature, without any adverse affects.

## Background

Victims of major trauma are considered to be at risk of hypothermia which often results in deleterious effects (including coagulopathy, cardiac arrhythmias, peripheral vasoconstriction, metabolic acidosis, compensatory increased oxygen requirements during rewarming, and impaired immune response) leading to an associated increased mortality and morbidity [1,2]. Up to 66% of trauma patients are reported to be hypothermic at the time of hospital admission [2]. Those patients with a core temperature lower than 34°C suffer a mortality rate up to 35% higher than euthermic patients [3-6].

At trauma sites it is common to transfuse red cells that have been stored and transported at 4°C and/or colloid/crystalloid fluids at ambient temperature. This administration of cold intravenous fluids has been identified as one of the main factors that contribute to the high incidence of hypothermia in the trauma population [1].

To prevent falls of temperature, in hospitals there are several mains powered devices available for warming fluids before infusion into patients. However at retrieval or accident sites electrical energy is generally only available in the form of batteries.

An alternative method of portable energy storage is in the form of latent heat in supercooled liquids. A liquid is supercooled when it remains in the liquid state at temperatures below its nominal solidification temperature. Many substances can exist as supercooled liquids, eg sodium acetate trihydrate (used in "heat packs") normally freezes at approximately 55°C, but can remain in the liquid state at temperatures below 10°C if it is kept free from impurities in a sealed container. When a supercooled liquid solidifies, it releases the latent heat of solidification and its temperature quickly approaches the nominal solidification temperature of the substance. This provides an ideal situation for warming intravenous fluids, providing the desired warming temperature corresponds to the solidification temperature of a latent heat storage material.

A fluid warmer based on latent heat could be a relatively simple and low-cost device. In its most basic form it may consist of some latent heat storage material, some IV tubing and an external casing.

This work aimed to determine if latent heat can be used to warm blood from its storage temperature (2°C – 6°C) to normal body temperature, this article describes the development of a latent heat fluid warmer and its in vitro testing.

## Methods

### Prototype construction

Prototype fluid warmers, consisting of (Gambro) infusion extension lines immersed in a latent heat storage material, with flexible or rigid external casings, were constructed. The latent heat storage material had a phase change temperature of 42°C, and was kept free from impurities to enable it to be supercooled. The prototype used to warm blood contained 0.85 litres of latent heat storage material.

### Red cell units

Fresh red cells (RC) stored in Optisol^®^, that were unsuitable for transfusion because they did not meet pre-determined criteria (such as the donor had visited a malarial risk area within the previous 4 months), were supplied by the Australian Red Cross Blood Service for in vitro tests. All these RC units were marked "Component for discard". These RC had a shelf life of 42 days from the day of collection and were kept under standard storage conditions (2°C – 6°C) in validated temperature monitored cold rooms until used for testing. The average volume of these RC units was 275 mL. RC with a range of storage times (1 to 42 days) were tested in the latent heat fluid warmer.

### Infusion flow rates

The infusion rate of red cells under normal clinical conditions (not warmed with latent heat) was sampled by timing infusions into patients in the Intensive and Critical Care Unit of Flinders Medical Centre. Red cells were delivered intravenously via 14, 16 or 20 gauge needles inserted into the patient's right cubital fossa or right hand, whilst being pressurised at 250 mmHg. All decisions affecting patient care (choice of needle gauge, insertion site, infusion pressure etc) were made by the staff of the Intensive and Critical Care Unit, and the investigators were merely observers.

### Latent heat prototype testing

Latent heat was used to warm 67 units of RC in 25 tests; 12 tests were conducted using 1 unit of RC, 11 tests were conducted using 4-units of RC, one test was conducted using 5 units of RC and the final test was conducted using 6-units of RC. RC were gently massaged by hand prior to testing, to mix the cells with clear fluid that had separated during storage.

**Figure 1:**
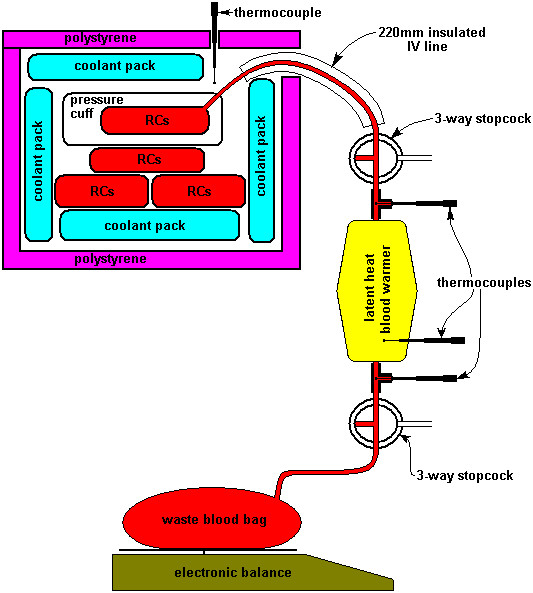
Test apparatus, 4 units of RC surrounded by coolant packs in a polystyrene box. One RC unit placed in a pressure cuff (which was inflated as required, to express the contents of the RC unit) and connected via a 220 mm insulated IV line and 3-way stopcock to the inlet of the blood warmer. A tube was connected from the blood warmer outlet via a 3-way stopcock to a waste bag on top of an electronic balance and the balance output was recorded at approximately 30-second intervals – for determining the outlet flow rate. 10 mL samples were withdrawn from the inlet stopcock when the RC from the unit first began flowing into the blood warmer, and from the outlet stopcock at the point where the unit was estimated to be half full and as the RC unit emptied. As each RC unit emptied, it was removed from the IV line and replaced with a full one (until all RC units in the polystyrene box had been used). K-type thermocouples, connected to a temperature logger, were placed in the blood warmer inlet and outlet ports, in the polystyrene box and in the latent heat storage material inside the blood warmer. Three-way stopcocks were placed immediately upstream from the thermocouple in the blood warmer inlet line and immediately downstream from the thermocouple in the blood warmer outlet line.

For each test 1, 4, 5 or 6 units of RC were surrounded by Australian Red Cross Blood Service validated insulated frozen coolant packs in a polystyrene box (<6°C), as shown in figure 1. K-type thermocouples, connected to a temperature logger, were placed in the fluid warmer inlet and outlet ports, in the polystyrene box and in the latent heat storage material inside the fluid warmer. Three-way stopcocks were placed immediately upstream from the thermocouple in the fluid warmer inlet line and immediately downstream from the thermocouple in the fluid warmer outlet line. A 220 mm insulated intravenous (IV) line was connected from the inlet stopcock to one of the RC units, which was placed inside a pressure cuff in the polystyrene box (the pressure cuff was inflated as required, to pressurise the RC units and express their contents). A tube was connected from the fluid warmer outlet to a waste bag on top of an electronic balance and the balance output was recorded at approximately 30-second intervals, for determining the outlet flow rate. 10 mL samples were withdrawn from the inlet stopcock when the RC from the unit first began flowing into the fluid warmer, and from the outlet stopcock at the point where the unit was estimated to be half full and as the RC unit emptied. As each RC unit emptied, it was removed from the IV line and replaced with a full one (until all RC units in the polystyrene box had been used).

A similar test protocol was used with a Cobe SpectraTherm (Lakewood, CO) dry heat mains powered fluid warmer (regulated drum temperature of 36.7°C). The dry heat blood warmer was used to warm 6 units of RC, with one unit being warmed in each test.

### Biochemical testing

In addition to the RC samples withdrawn during fluid warmer testing, 10 mL samples were extracted from 4 RC units kept in storage (2°C – 6°C) for their lifetimes (42 days), on storage days 2, 3, 6, 8, 9, 10, 13, 16, 22, 30, 34, 37 and 42. These samples were centrifuged and supernatant extracted (as described below) while they were cold.

Plasma potassium (K), lactate dehydrogenase (LD) and haemoglobin (Hb) levels were determined to assess red cell damage and level of haemolysis occurring either during normal storage conditions (2°C – 6°C) or following warming through the approved dry heat blood warmer or the prototype.

All samples were centrifuged at an accelerating force of 2050 g for 4 minutes, after which time 500 μL of supernatant was removed and analyte concentrations were determined:

Potassium (mmol/L) was measured with a Roche modular ISE1800 analyser.

Lactate dehydrogenase (units/L) and a serum index, equivalent to haemoglobin concentration (mg/dl) were determined with a Roche modular P800 analyser.

## Results

### Infusion flow rates

For the limited number of timed infusions (table 1) using 14 or 16 gauge needles and pressurised at 250 mmHg, the average infusion rate was approximately 50 mL/minute. The infusion rate of RC is highly dependent on factors such as needle gauge (Poiseuille's law) and venous access site, therefore in a particular infusion, the actual rate of infusion may vary from the rates measured in this case.

**Table 1:** Rapid infusion flow rates

Needle Gauge	No of infusions	Average flow rate (mL/min)
14	2	54
16	2	43
20	1	24

### Fluid warmer testing

**Figure 2:**
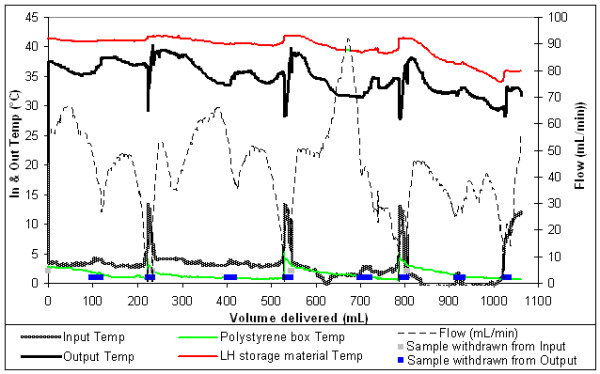
Latent heat warming 4 units of RC, temperatures & flow graphed against volume.

**Figure 3:**
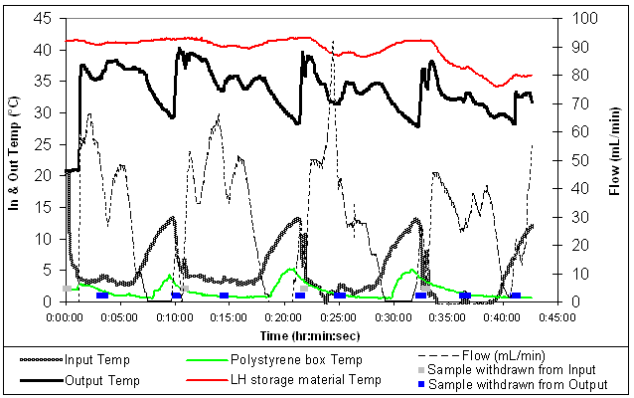
Latent heat warming 4 units of RC, temperatures & flow graphed against time.

Most of the RC warmed with latent heat were in batches of 4 – as would occur if a retrieval patient required approximately 1100 mL of RC. Figure 2 shows typical results for thses tests, four RC units were warmed from an average inlet temperature of 3°C to an average of 35°C at flow rates of 30–60 mL/minute. Halting the flow for approximately 3 minutes, while empty units were replaced, provided an opportunity to determine the extent to which the fluid warmer output was temperature regulated. Figure 3 shows the same test results as figure 2, graphed against time instead of volume, demonstrating the limited extent of temperature overshoot that occurred whilst fluid flow through the prototype was halted; neither the RC nor the latent heat storage material exceeded the phase change temperature of 42°C.

The output of the latent heat prototype fluid warmer did decrease over time (as the release of latent heat decreased).

**Figure 4:**
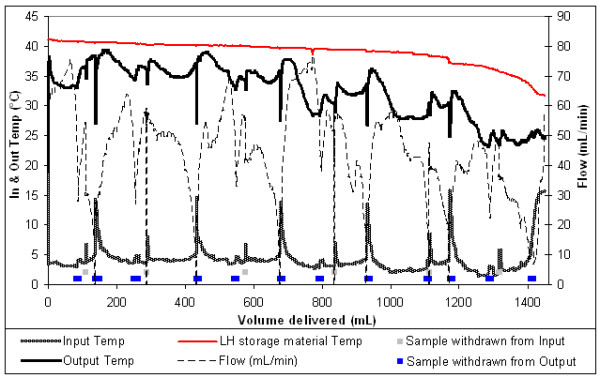
Latent heat warming 6 units of RC.

Six RC units were also warmed from approximately 4°C to an average of 32°C (figure 4). The rapid decline in latent heat storage material temperature at the end of this test indicates that the maximum number of RC units that can be realistically warmed from 4°C with 0.85 litres of the latent heat storage material is 6.

**Figure 5:**
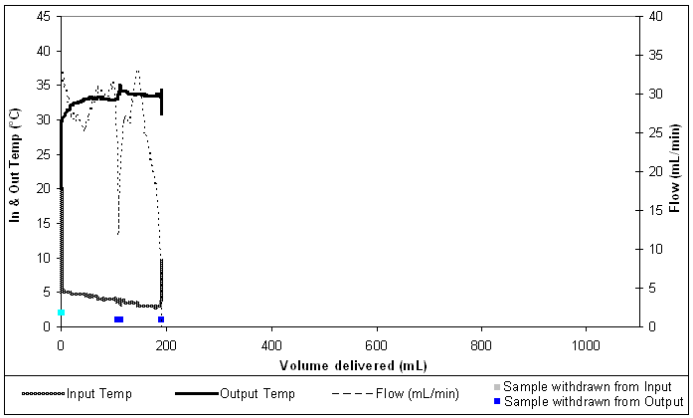
Dry heat blood warmer warming 1 unit of RC.

The dry heat blood warmer (figure 5) warmed single units of RC from an average 3°C to 33°C.

### Biochemical testing

**Figure 6:**
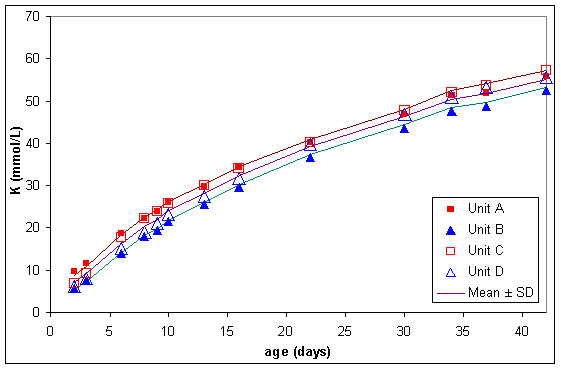
Extracellular potassium (K) in 4 red cell units during storage.

**Figure 7:**
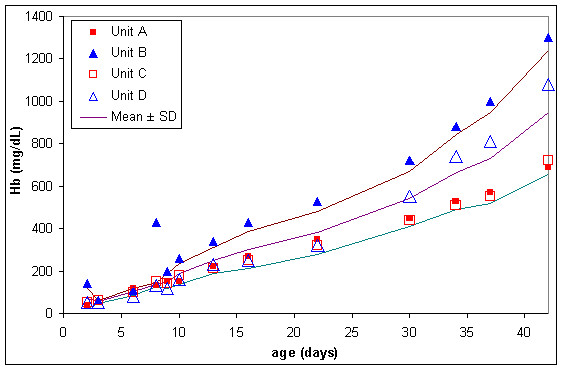
**Extracellular haemoglobin (Hb) in 4 red cell units during storage**. Result for unit B on day 8 appears to be an anomaly, so has not been included in mean or SD calculation.

**Figure 8:**
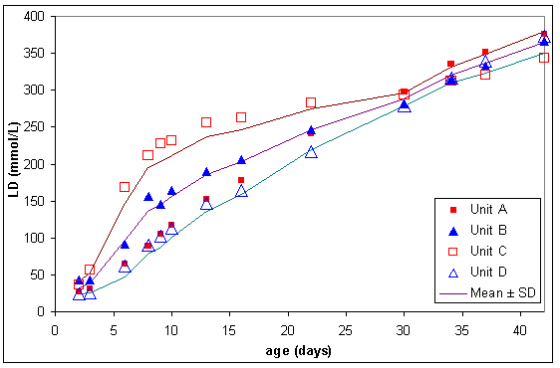
Extracellular lactate dehydrogenase (LD) in 4 red cell units during storage.

The normal lesion occurring in stored blood was quite severe. In the four RC units kept in storage mean extracellular potassium concentration (figure 6) increased from approximately 7 to 55 mmol/L from storage day 2 to day 42, plasma haemoglobin (figure 7) increased from approximately 70 to 1000 mg/dL and lactate dehydrogenase (figure 8) increased from approximately 30 to 360 units/l.

**Figure 9:**
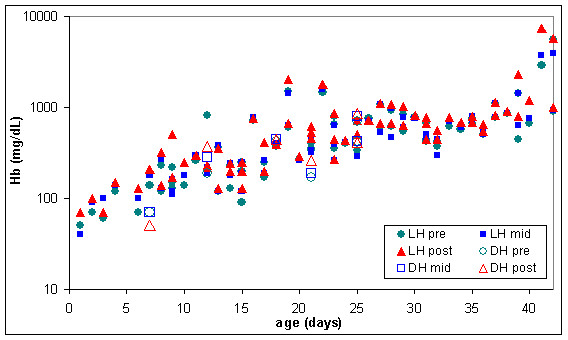
**Extracellular haemoglobin (Hb) in pre, mid & post warmed RC warmed with latent heat or the dry heat blood warmer (n = 67)**. LH pre = Latent heat prototype, sample extracted from inlet line (before warming). LH mid = Latent heat prototype, sample extracted from outlet line midway through RC unit (warmed). LH post = Latent heat prototype, sample extracted from outlet line at the end of the RC unit (warmed). DH pre = Dry heat blood warmer, sample extracted from inlet line (before warming). DH mid = Dry heat blood warmer, sample extracted from outlet line midway through RC unit (warmed). DH post = Dry heat blood warmer, sample extracted from outlet line at the end of the red RC (warmed).

**Figure 10:**
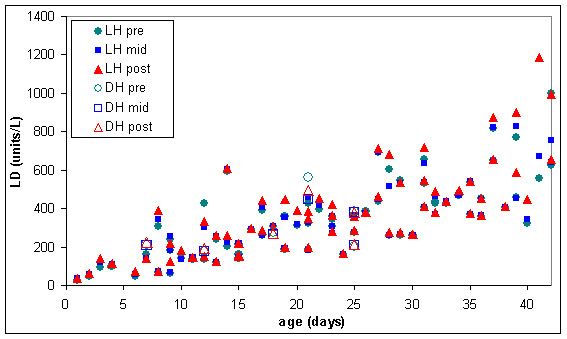
**Extracellular lactate dehydrogenase (LD) in pre, mid & post warmed RC warmed with latent heat or the dry heat blood warmer (n = 67)**. LH pre = Latent heat prototype, sample extracted from inlet line (before warming). LH mid = Latent heat prototype, sample extracted from outlet line midway through RC unit (warmed). LH post = Latent heat prototype, sample extracted from outlet line at the end of the RC unit (warmed). DH pre = Dry heat blood warmer, sample extracted from inlet line (before warming). DH mid = Dry heat blood warmer, sample extracted from outlet line midway through RC unit (warmed). DH post = Dry heat blood warmer, sample extracted from outlet line at the end of the red RC (warmed).

In some of the warmed RC units the lesion appears to be much worse, as indicated in figures 9 and 10 where extracellular haemoglobin concentration was well over 1000 mg/dL and lactate dehydrogenase was over 800 units/L in some RC units before warming.

It was found that there was usually considerable supernatant in RC samples extracted before warming, and there was much less supernatant in samples extracted from the outlet mid way through RC units and even less supernatant in samples extracted from the outlet at the end of RC units. This was probably an indication that cells had settled towards the bottom of RC units, so the first portion expressed from RC units was the leanest in cells and the last portion expressed was the richest in cells.

**Figure 11:**
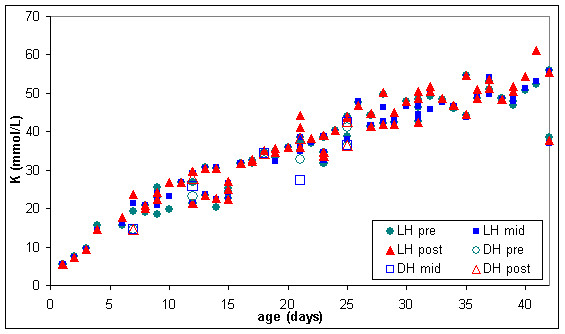
**Extracellular potassium (K) in pre, mid & post warmed RC warmed with latent heat or the dry heat blood warmer (n = 67)**. LH pre = Latent heat prototype, sample extracted from inlet line (before warming). LH mid = Latent heat prototype, sample extracted from outlet line midway through RC unit (warmed). LH post = Latent heat prototype, sample extracted from outlet line at the end of the RC unit (warmed). DH pre = Dry heat blood warmer, sample extracted from inlet line (before warming). DH mid = Dry heat blood warmer, sample extracted from outlet line midway through RC unit (warmed). DH post = Dry heat blood warmer, sample extracted from outlet line at the end of the red RC (warmed).

Measurements of extracellular potassium (figures 6 and 11) were very similar in stored and warmed units of red cells. Plasma haemoglobin (figures 7 and 9) and lactate dehydrogenase (figures 8 and 10) exhibited larger variations in the 67 warmed units of RC than in the 4 units of RC that remained in storage. This large spread of results was evident in the "pre" samples extracted before warming as well as the "mid" and post" samples extracted after warming (figures 9 and 10) indicating that most of the variation in haemoglobin and lactate dehydrogenase concentration was due to factors other than warming of the RC units.

**Figure 12:**
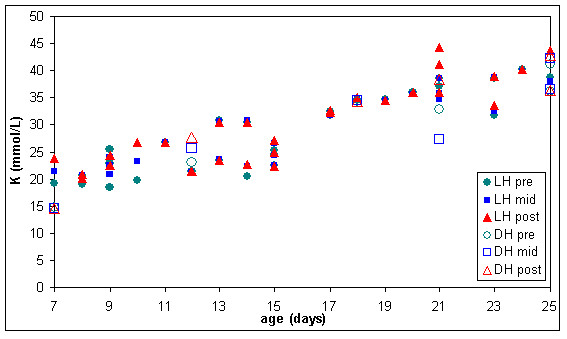
**Extracellular potassium (K) in the subset of pre, mid & post warmed RC (tested between day 7 & 25 of storage, with initial haemoglobin concentration between 70 & 700 mg/dL) warmed with latent heat (n = 28) or the dry heat blood warmer (n = 6)**. LH pre = Latent heat prototype, sample extracted from inlet line (before warming). LH mid = Latent heat prototype, sample extracted from outlet line midway through RC unit (warmed). LH post = Latent heat prototype, sample extracted from outlet line at the end of the RC unit (warmed). DH pre = Dry heat blood warmer, sample extracted from inlet line (before warming). DH mid = Dry heat blood warmer, sample extracted from outlet line midway through RC unit (warmed). DH post = Dry heat blood warmer, sample extracted from outlet line at the end of the red RC (warmed).

**Figure 13:**
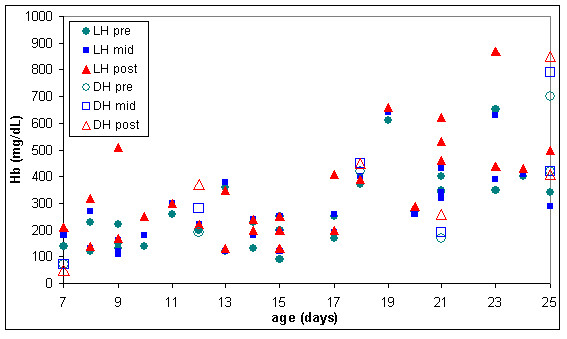
**Extracellular haemoglobin (Hb) in the subset of pre, mid & post warmed RC (tested between day 7 & 25 of storage, with initial haemoglobin concentration between 70 & 700 mg/dL) warmed with latent heat (n = 28) or the dry heat blood warmer (n = 6)**. LH pre = Latent heat prototype, sample extracted from inlet line (before warming). LH mid = Latent heat prototype, sample extracted from outlet line midway through RC unit (warmed). LH post = Latent heat prototype, sample extracted from outlet line at the end of the RC unit (warmed). DH pre = Dry heat blood warmer, sample extracted from inlet line (before warming). DH mid = Dry heat blood warmer, sample extracted from outlet line midway through RC unit (warmed). DH post = Dry heat blood warmer, sample extracted from outlet line at the end of the red RC (warmed).

**Figure 14:**
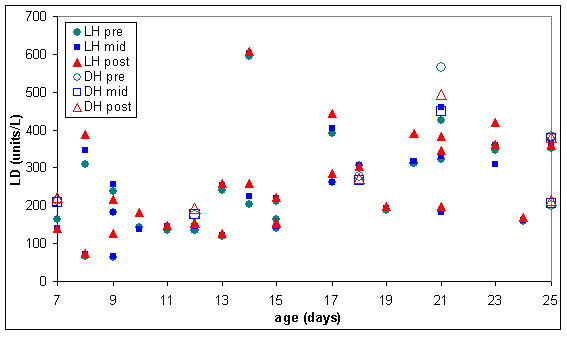
**Extracellular lactate dehydrogenase (LD) in the subset of pre, mid & post warmed RC (tested between day 7 & 25 of storage, with initial haemoglobin concentration between 70 & 700 mg/dL) warmed with latent heat (n = 28) or the dry heat blood warmer (n = 6)**. LH pre = Latent heat prototype, sample extracted from inlet line (before warming). LH mid = Latent heat prototype, sample extracted from outlet line midway through RC unit (warmed). LH post = Latent heat prototype, sample extracted from outlet line at the end of the RC unit (warmed). DH pre = Dry heat blood warmer, sample extracted from inlet line (before warming). DH mid = Dry heat blood warmer, sample extracted from outlet line midway through RC unit (warmed). DH post = Dry heat blood warmer, sample extracted from outlet line at the end of the red RC (warmed).

The six units of RC warmed with the dry heat blood warmer were aged between 7 and 25 days and had extracellular haemoglobin concentrations between 70 and 700 mg/dL before warming. To compare the effect of warming RC with latent heat and the dry heat blood warmer, a subset of the results from figures 9 to 11 was used. This subset consisted of all the biochemical test results obtained for RC units warmed between days 7 and 25 of storage and with extracellular haemoglobin concentrations between 70 and 700 mg/dL, before warming. The subset included 28 RC units warmed with latent heat as well as the 6 RC units warmed with the dry heat blood warmer. Figures 12 to 14 show the biochemical test results for this subset, and indicate that changes in extracellular potassium, haemoglobin and lactate dehydrogenase concentrations during RC warming were similar for both the latent heat prototype and dry heat blood warmers.

The means and standard deviations of extracellular potassium, haemoglobin and lactate dehydrogenase concentrations in this subset (table 2) indicate that the concentrations of all analytes generally increased in RC after warming, but the increase is similar for both methods of warming employed here.

**Table 2:** Means and standard deviations of extracellular potassium, haemoglobin and lactate dehydrogenase concentrations in RC units warmed with latent heat (n = 28) or the dry heat blood warmer (n = 6) between day 7 & 25 of storage, with initial haemoglobin concentration between 70 & 700 mg/dL

		Sample from inlet line (before warming)		Sample from outlet line (midway through RC unit)		Sample from outlet line (at RC unit end)	
				
		Latent heat	Dry heat	Latent heat	Dry heat	Latent heat	Dry heat
Potassium (mmol/L)	Mean	29	30	29	30	30	32
	Standard deviation	7.2	10	6.6	10	7.4	10
Haemoglobin (mg/dL)	Mean	267	328	275	367	349	398
	Standard deviation	139	231	140	251	183	264
Lactate Dehydrogenase (units/L)	Mean	240	302	244	282	262	296
	Standard deviation	120	148	124	109	127	119

## Discussion

Flow rates of ambient temperature saline (pressurised at 300 mmHg) through IV delivery systems, including a fluid warmer and 14 gauge cannula have been found to be between 225 and 446 mL/minute, depending upon the type of fluid warmer used [7]. The flow rates sampled in the present study in actual clinical situations were significantly lower. Reasons for the lower flow rate in the present study include: 250 mmHg used instead of 300 mmHg, the viscosity of blood is much higher than that of saline and flow restrictions due to venous access sites.

Red cells have previously been heated to 44.7°C for 30 minutes with no damage reported [8], and it has also been shown that incubation at 45°C for up to 1 hour does not cause significant haemolysis [9]. Therefore damage due to heating red cells to 42°C with the latent heat storage material in the present study was not expected. Since latent heat is only released at or below the solidification temperature there could be no possibility of overheating due to using the latent heat of solidification as an energy source for warming blood.

Plasma potassium, lactate dehydrogenase and haemoglobin were used in this study to indicate RC damage, because they have previously been shown to be accurate markers of haemolysis [10].

In general extracellular concentrations of these analytes were higher in samples after warming than before warming in the present study. This may indicate damage as a result of warming, but could also be a function of sampling. As the amount of supernatant was greatest in the first (unwarmed) samples and least in the last (warmed) samples from RC units, the amount of cells in extracted samples was least in the first samples and greatest in the last samples from the RC units. The amount of extracellular potassium, haemoglobin and lactate dehydrogenase could be expected to be related to the amount of cells in the samples. Therefore the concentration of these analytes could be expected to be lowest in the first ("pre") samples extracted from RC units and highest in the last ("post") samples extracted from RC units whether units were warmed or not.

Although there was considerable variation in RC units before warming in this study, when the biochemical test results for units of similar age and initial condition were compared for the latent heat prototype and the dry heat blood warmer similar results were obtained for both warming methods. Overall the results of the biochemical tests for the latent heat fluid warmer, showed no signs of increased cellular damage compared to red cells passed through the clinically approved dry heat blood warmer.

It should be noted that the severity of the storage lesion of blood found in this study may not be an indication of typical storage lesion, because the RC units used in this study had been declared unsuitable for transfusion.

The further development of the use of latent heat as a blood warming modality will depend upon the willingness of medical device manufacturers to explore this field.

## Conclusion

The feasibility of warming blood and other intravenous fluids with a latent heat storage material has been demonstrated. A low-cost portable non-electrically powered fluid warmer using latent heat storage may be used to significantly warm cold blood or intravenous fluids to near body temperature with minimal risk of cellular damage and haemolysis.

The big advantage of warming IV fluids using the latent heat of fusion of a solidifying liquid is that heat is released at the solidification temperature, which is inherently temperature limiting. Consequently the risk of overheating fluids with a latent heat IV fluid warmer is negligible. Testing reported in this paper found that the warm blood outlet temperature from a latent heat fluid warmer did not exceed the phase change temperature of the latent heat storage material, even during times of flow cessation.

## Abbreviations

RC red cells

K potassium

LD lactate dehydrogenase

Hb haemoglobin

LH latent heat prototype blood warmer

DH dry heat blood warmer

SD standard deviation

g acceleration due to gravity

IV intravenous

pre sample extracted from blood warmer inlet line (before warming)

mid sample extracted from blood warmer outlet line midway through RC unit (warmed)

post sample extracted from blood warmer outlet line at the end of the RC unit (warmed)

n number of RC units tested

## Competing interests

Both authors have been named as inventors in 2 patent applications lodged by Flinders Biomedical Engineering, for biological fluid warming.

## Authors' contributions

MPM co-conceived the study, constructed the prototype fluid warmers, carried out all fluid warming and infusion flow rate tests, compiled biochemical test data and drafted the manuscript.

DR co-conceived the study, arranged the supply of blood from Red Cross, extracted samples from blood units during storage, compiled biochemical test data and drafted the manuscript.

## Pre-publication history

The pre-publication history for this paper can be accessed here:


